# Priming astrocytes with TNF enhances their susceptibility to *Trypanosoma cruzi* infection and creates a self-sustaining inflammatory milieu

**DOI:** 10.1186/s12974-017-0952-0

**Published:** 2017-09-06

**Authors:** Andrea Alice Silva, Rafael Rodrigues Silva, Daniel Gibaldi, Rafael Meyer Mariante, Jessica Brandão dos Santos, Isabela Resende Pereira, Otacílio Cruz Moreira, Joseli Lannes-Vieira

**Affiliations:** 1 Laboratório de Biologia das Interações, Instituto Oswaldo Cruz – Fiocruz, Av. Brasil 4365, Rio de Janeiro, RJ 21040-360 Brazil; 2 Laboratório Multidisciplinar de Apoio à Pesquisa em Nefrologia e Ciências Médicas, Departamento de Patologia, Faculdade de Medicina, Universidade Federal Fluminense, Rua Marquês do Paraná, 303, Niterói, RJ 24033-900 Brazil; 3 Laboratório de Biologia Estrutural IOC/Fiocruz, Av. Brasil 4365, Rio de Janeiro, RJ 21040-360 Brazil; 4 Laboratório de Biologia Molecular e Doenças Endêmicas, IOC/Fiocruz, Av. Brasil 4365, Rio de Janeiro, RJ 21040-360 Brazil; 5 Laboratório de Doença de Chagas, Escola de Farmácia, Universidade Federal de Ouro Preto, Campus Morro do Cruzeiro s/no, Ouro Preto, MG 35400-000 Brazil; 6 Laboratório de Hematologia, Departamento de Patologia, Faculdade de Medicina, Universidade Federal Fluminense, Rua Marquês do Paraná, 303, Niterói, RJ 24033-900 Brazil

**Keywords:** Tumor necrosis factor, Chagas disease, Pentoxifylline, Anti-TNF antibody Infliximab, TNFR1

## Abstract

**Background:**

In conditions of immunosuppression, the central nervous sty 5ystem (CNS) is the main target tissue for the reactivation of infection by *Trypanosoma cruzi*, the causative agent of Chagas disease. In experimental *T. cruzi* infection, interferon gamma (IFNγ)^+^ microglial cells surround astrocytes harboring amastigote parasites. In vitro, IFNγ fuels astrocyte infection by *T. cruzi*, and IFNγ-stimulated infected astrocytes are implicated as potential sources of tumor necrosis factor (TNF). Pro-inflammatory cytokines trigger behavioral alterations. In *T. cruzi*-infected mice, administration of anti-TNF antibody hampers depressive-like behavior. Herein, we investigated the effects of TNF on astrocyte susceptibility to *T. cruzi* infection and the regulation of cytokine production.

**Methods:**

Primary astrocyte cultures of neonatal C57BL/6 and C3H/He mice and the human U-87 MG astrocyte lineage were infected with the Colombian *T. cruzi* strain. Cytokine production, particularly TNF, and TNF receptor 1 (TNFR1/p55) expression were analyzed. Recombinant cytokines (rIFNγ and rTNF), the anti-TNF antibody infliximab, and the TNFR1 modulator pentoxifylline were used to assess the in vitro effects of TNF on astrocyte susceptibility to *T. cruzi* infection. To investigate the role of TNF on CNS colonization by *T. cruzi*, infected mice were submitted to anti-TNF therapy.

**Results:**

rTNF priming of mouse and human astrocytes enhanced parasite/astrocyte interaction (i.e., the percentage of astrocytes invaded by trypomastigote parasites and the number of intracellular parasite forms/astrocyte). Furthermore, *T. cruzi* infection drove astrocytes to a pro-inflammatory profile with TNF and interleukin-6 production, which was amplified by rTNF treatment. Adding rTNF prior to infection fueled parasite growth and trypomastigote egression, in parallel with increased TNFR1 expression. Importantly, pentoxifylline inhibited the TNF-induced increase in astrocyte susceptibility to *T. cruzi* invasion. In *T. cruzi*-infected mice, anti-TNF therapy reduced the number of amastigote nests in the brain.

**Conclusions:**

Our data implicate TNF as a promoter of *T. cruzi* invasion of mouse and human astrocytes. Moreover, the TNF-enriched inflammatory milieu and enhanced TNFR1 expression may favor TNF signaling, astrocyte colonization by *T. cruzi* and egression of trypomastigotes. Therefore, in *T. cruzi* infection, a self-sustaining TNF-induced inflammatory circuit may perpetuate the parasite cycle in the CNS and ultimately promote cytokine-driven behavioral alterations.

**Electronic supplementary material:**

The online version of this article (doi:10.1186/s12974-017-0952-0) contains supplementary material, which is available to authorized users.

## Background

Astrocytes are heterogeneous cells that are involved in maintaining the homeostatic environment in the central nervous system (CNS) [1]. They are the most abundant cell type in the brain and play an important role in several infectious and inflammatory diseases [2–5]. Like microglia and neurons, astrocytes can produce inflammatory mediators such as chemokines and cytokines, including the potent pro-inflammatory tumor necrosis factor (TNF), in response to intrinsic and extrinsic insults [6, 7]. TNF binds to two different receptors, TNFR1 (p55) and TNFR2 (p75), and mediates various biological responses in cells bearing these receptors [8, 9]. Astrocytes constitutively express *Tnfr1* mRNA and nearly undetectable *Tnfr2* mRNA, neither of which are upregulated after exposure to interferon gamma (IFNγ) or TNF [10]. TNF and interleukin (IL)-6, another pro-inflammatory cytokine, are positively correlated with the rate of neuronal apoptosis in the CNS and with neurodegeneration [11, 12]. Increased TNF levels have been associated with neuropathology in animal models of sleeping sickness, a human disease induced by infection with the extracellular parasite *Trypanosoma brucei*, and malaria, caused by *Plasmodium* sp. [13–15]. Indeed, TNF dysregulation has been associated with blood-brain barrier abnormalities and the initiation of CNS inflammation and pathology in non-infectious and infectious diseases [16]. However, the effect of TNF in the CNS during *Trypanosoma cruzi* infection is unclear. This intracellular protozoan parasite is the etiologic agent of Chagas disease, a neglected tropical disease characterized by a systemic inflammatory response enriched in TNF in chronic patients [17, 18] and experimental models [19].

In the acute phase of *T. cruzi* infection, particularly in infants and susceptible experimental mice, intense meningoencephalitis and accumulation of amastigote forms of the parasite inside glial cells are common, but CNS inflammation and parasitism are drastically reduced in the chronic phase [20–29]. Nevertheless, in chronic *T. cruzi* infection, neurological involvement is observed in 75–90% of human immunodeficiency virus (HIV)-coinfected patients and in individuals immunocompromised by cancer, suppressive treatment, organ transplantation, or malnourishment [30, 31]. In these situations, the most common findings are neurological abnormalities, recrudescence of parasitemia, and parasite detection in the brain tissue and cerebrospinal fluid [21, 24, 30]. In early diagnosed patients, the signs of CNS impairment disappear after treatment with the trypanocidal drug benznidazole [22–24]. In chronically infected individuals, reactivation of CNS parasitism may occur via the re-entry of blood circulating parasites; CNS invasion by inflammatory cells carrying parasite, likewise the macrophages carrying HIV by a Trojan horse mechanism [32]; or increase of low-grade CNS parasitism. In chronically *T. cruzi*-infected C3H/He mice undergoing immunosuppression, reactivation of infection with numerous and large parasite nests and inflammation is restricted to the CNS and occurs in the absence of parasitemia, supporting the existence of low-grade parasite persistence in CNS-resident cells [27]. Nevertheless, the biological processes that may favor *T. cruzi* persistence in the CNS cells remain unclear. Recently, we showed that in chronically infected C3H/He mice, most *T. cruzi*-bearing astrocytes are located near interferon gamma (IFNγ)^+^ Iba1^+^ microglial cells, in the absence of neuroinflammation [29]. The susceptibility of mouse primary astrocyte cultures to *T. cruzi* infection is increased by IFNγ, and the anti-TNF antibody infliximab abolished this IFNγ-induced effect. Therefore, IFNγ-stimulated infected astrocytes are potential sources of TNF, which may facilitate astrocyte infection [29]. In the present study, we used primary mouse astrocyte cultures and the human U-87 MG lineage to investigate whether *T. cruzi* infection triggers TNF production and to explore the biological effects of TNF on parasite/astrocyte interaction regarding the *T. cruzi* cycle (invasion by trypomastigotes, growth of intracellular forms, and egression of trypomastigotes) and cytokine profile. Furthermore, we treated *T. cruzi*-infected mice with anti-TNF antibody to investigate whether TNF indeed affects CNS parasitism.

## Methods

### Primary mouse astrocyte cultures

For in vitro experiments, primary mouse astrocyte cell cultures were obtained from the cerebral cortex of 1-day-old C57BL/6 (H-2^b^) and C3H/He (H-2^K^) mice, as described previously [33]. Briefly, mice were decapitated, and the meninges were removed. Cortices were then isolated and minced, and a single-cell suspension was obtained by mechanical dissociation. The cells from each brain were seeded into a 25-cm^2^ culture flask pre-coated with poly-ornithine (0.1 mg/mL; Sigma-Aldrich, St. Louis, MO, USA) and maintained in DMEM/F-12 medium (Gibco, Gaithersburg, MD, USA) containing 10% fetal bovine serum (FBS; Gibco, Gaithersburg, MD, USA) at 37 °C in a 95% humid atmosphere with 5% CO_2_. Microglia and other non-adherent cells were removed by gently shaking the culture flasks. Astrocytes from 7 to 10 days of culture were detached with 0.25% trypsin +0.02% EDTA in phosphate-buffered saline (PBS), pH 7.2–7.4, and plated at a density of 10^5^ cells per 13-mm-diameter poly-ornithine-coated coverslip in 24-well plates (NUNC, Roskilde, Denmark). Cells were cultured for 24 h to allow adhesion to coverslips. Details of each experiment are provided in the figure legends.

### Characteristics of primary mouse astrocyte cultures

The cell composition of primary astrocyte cultures was determined by indirect immunofluorescence using purified mouse anti-glial fibrillary acidic protein (GFAP) (Invitrogen, Carlsbad, CA, USA) and FITC-conjugated anti-mouse immunoglobulin secondary antibody (Dako, Carpinteria, CA, USA). Microglia were revealed by surface CD11b expression using PECy7-conjugated anti-mouse CD11b monoclonal antibody (eBioscience, San Diego, CA, USA). After cultivation, cells that adhered to coverslips were washed with warm PBS and fixed with methanol. To block nonspecific binding, the cells were incubated for 30 min with normal goat serum diluted 1:50 in 0.1% sodium azide/PBS and then stained for GFAP and CD11b. Negative controls were performed by omitting the primary antibodies. RAW 264.7 mouse macrophage cells (TIB-71™, ATCC®, Manassas, VA, USA) cultured in parallel served as a positive control for CD11b expression [29]. The coverslips were mounted onto slides with Prolong Gold Antifade reagent containing the fluorescent nuclear dye DAPI (Life Technologies, Carlsbad, CA, USA), which stains cell nuclei. The samples were then examined on a fluorescence microscope coupled to a digital camera DFC300FX (Leica, Wetzlar, Germany). The acquired images were analyzed using the IM50 Image Manager software (Leica, Wetzlar, Germany).

### L-929 fibroblast culture and TNF treatment

The fibroblast cell line L-929 (CCL-1™, ATCC®, Manassas, VA, USA), originated from a C3H/An mouse, was kindly provided by Dr. Maria de Nazaré Correia Soeiro (Laboratory of Cellular Biology, IOC, Fiocruz). Cells were cultivated in RPMI medium supplemented with 10% FBS and 1% penicillin-streptomycin (Sigma-Aldrich, St. Louis, MO, USA) and maintained at 37 °C in a 95% humid atmosphere with 5% CO_2_. Cells were detached with 0.25% trypsin +0.02% EDTA in PBS, washed, plated at a density of 5 × 10^4^ cells per 13-mm-diameter coverslip in 24-well plates (NUNC, Roskilde, Denmark), and incubated for 24 h to allow adhesion. Cultures were then washed with warm PBS, and the medium was replaced. To assess the effects of TNF in cells other than astrocytes, the fibroblast cultures were either left untreated or were treated with TNF (1 ng/mL) for 2 h and then infected as described for astrocytes.

### Human astrocyte lineage

Human U-87 MG glioblastoma cells were purchased from Banco de Células do Rio de Janeiro (BCRJ, Rio de Janeiro, RJ, Brazil). Cells were cultivated in DMEM medium supplemented with 10% FBS and 1% penicillin-streptomycin (Sigma-Aldrich, St. Louis, MO, USA) and maintained at 37 °C in a 95% humid atmosphere with 5% CO_2_. Cell culture dissociation was performed by replacing medium by 0.25% trypsin +0.02% EDTA in PBS. Cells were plated at a density of 10^5^ cells per 13-mm-diameter poly-ornithine-coated coverslips in 24-well plates (NUNC, Roskilde, Denmark). Details of each experiment are provided in the figure legends.

### Parasites for in vitro infection of cells

Trypomastigote forms of the Colombian Type I *T. cruzi* strain [34] were obtained from cultures of the Vero cell line (CCL81™ ATCC®, Manassas, VA, USA) maintained in 25-cm^2^ cultivation bottles (Falcon, Austin, TX, USA) using RPMI-1640/HEPES (Sigma-Aldrich, St. Louis, MO, USA). After 5 to 7 days in culture, the trypomastigote-containing supernatants were centrifuged (20 min, 1400×*g* at 4 °C); the parasites concentrated in the pellet were then resuspended, counted in a Neubauer chamber, and added to astrocyte or fibroblast cultures. Depending on the experimental design, we used a multiplicity of infection (MOI) of 1:1 or 10:1 (parasite/cell) and interaction periods of 4 or 24 h.

### Treatment of astrocytes with recombinant IFNγ and TNF in vitro

**Fig. 1:**
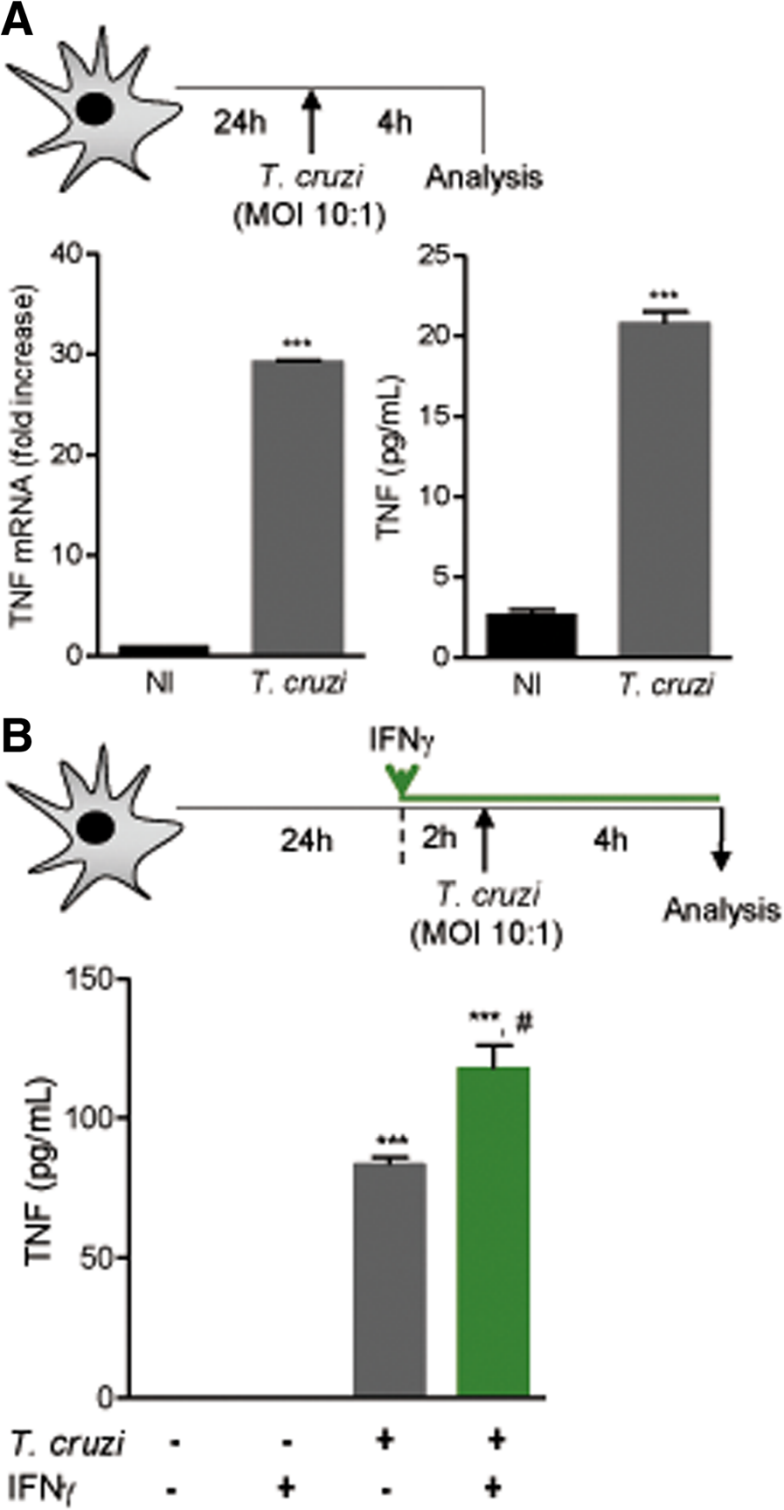
TNF is produced by *Trypanosoma cruzi-*infected astrocytes and is upregulated upon prior stimulation with rIFNγ. **a** C57BL/6 primary astrocyte cultures were infected with trypomastigote forms of the Colombian strain (MOI of 10:1) and analyzed 4 h later. *Tnf* mRNA expression was detected by RT-qPCR and TNF levels in the supernatants by ELISA. *T. cruzi* infection increased TNF production by astrocytes. **b** Primary astrocyte cultures were pre-treated with rIFNγ, infected with *T. cruzi* at a MOI of 10:1, and supernatants collected 4 h later. TNF levels in the supernatants were evaluated by ELISA. Pre-treatment with rIFNγ upregulates TNF production by *T. cruzi*-infected astrocytes. Data are shown as the means ± SEM of triplicates assays. ^***^ *p* < 0.001 for non-infected (NI) vs. *T. cruzi*-infected astrocytes. ^#^ *p* < 0.05 for untreated (NT) vs. rIFNγ-treated infected astrocytes

**Fig. 2:**
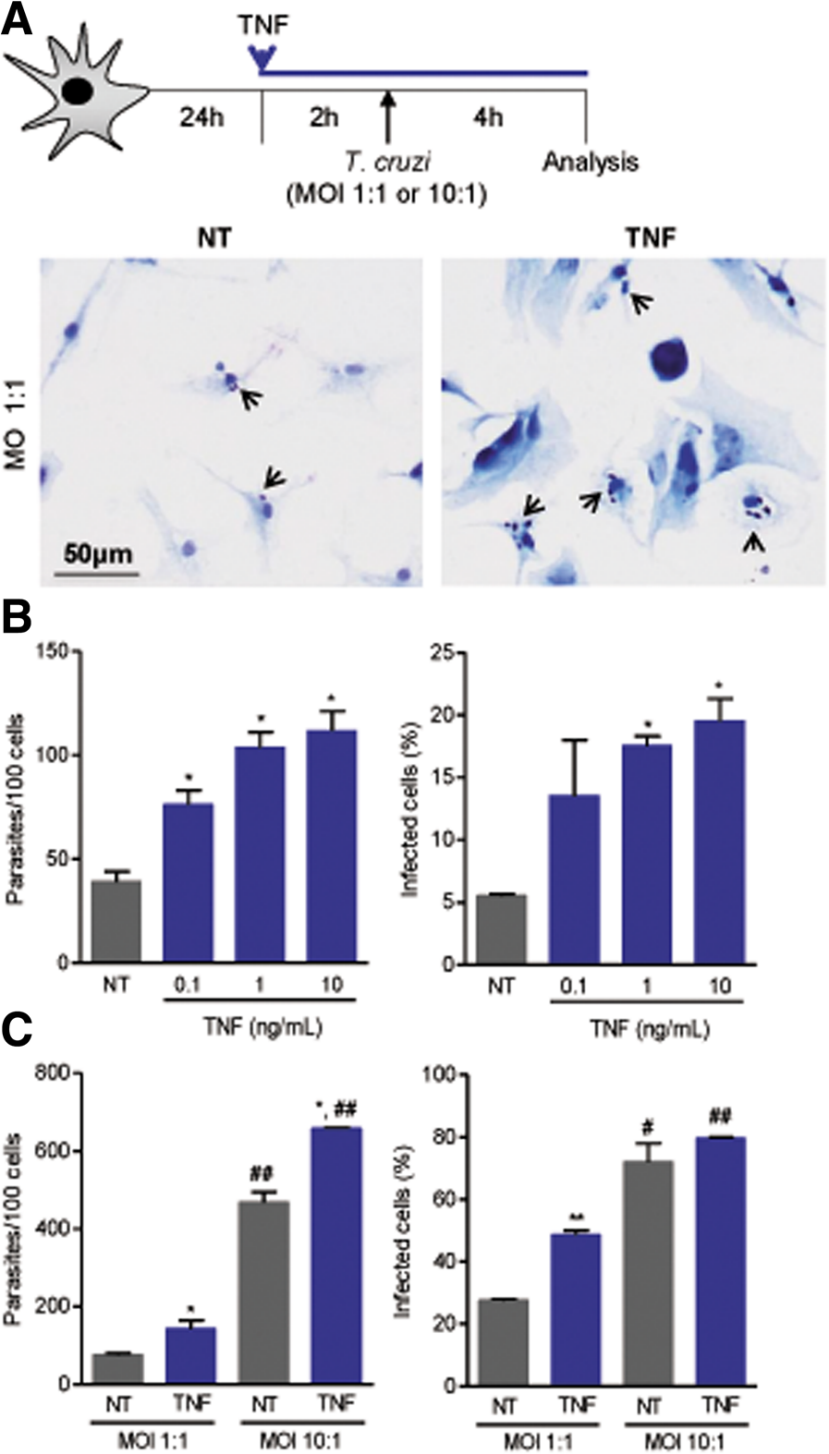
TNF pre-treatment enhances the infection of astrocyte cultures by *Trypanosoma cruzi*. **a** C57BL/6 primary astrocyte cell cultures were pre-treated with rTNF, infected at MOIs of 1:1 and 10:1, and analyzed 4 h postinfection. Giemsa staining revealed intracellular forms of *T. cruzi* (*arrows*) in untreated (NT) and TNF-treated infected astrocytes. **b** Astrocyte cultures were pre-treated with rTNF (0.1, 1, 10 ng/mL final concentrations) and infected with *T. cruzi* (MOI 1:1, 4 h), and the number of parasites per 100 cells and the percentage of infected cells were analyzed. **c** Number of parasites per 100 cells and the percentage of infected cells were analyzed after infection at MOIs of 1:1 and 10:1 and pre-treatment with rTNF at 1 ng/mL. Data are presented as the means ± SEM of triplicates. ^*^ *p* < 0.05 and ^**^ *p* < 0.01 for untreated (NT) vs. rTNF pre-treated astrocytes. ^#^ *p* < 0.05 and ^##^ *p* < 0.01 MOI of 1:1 vs. MOI of 10:1

**Fig. 3:**
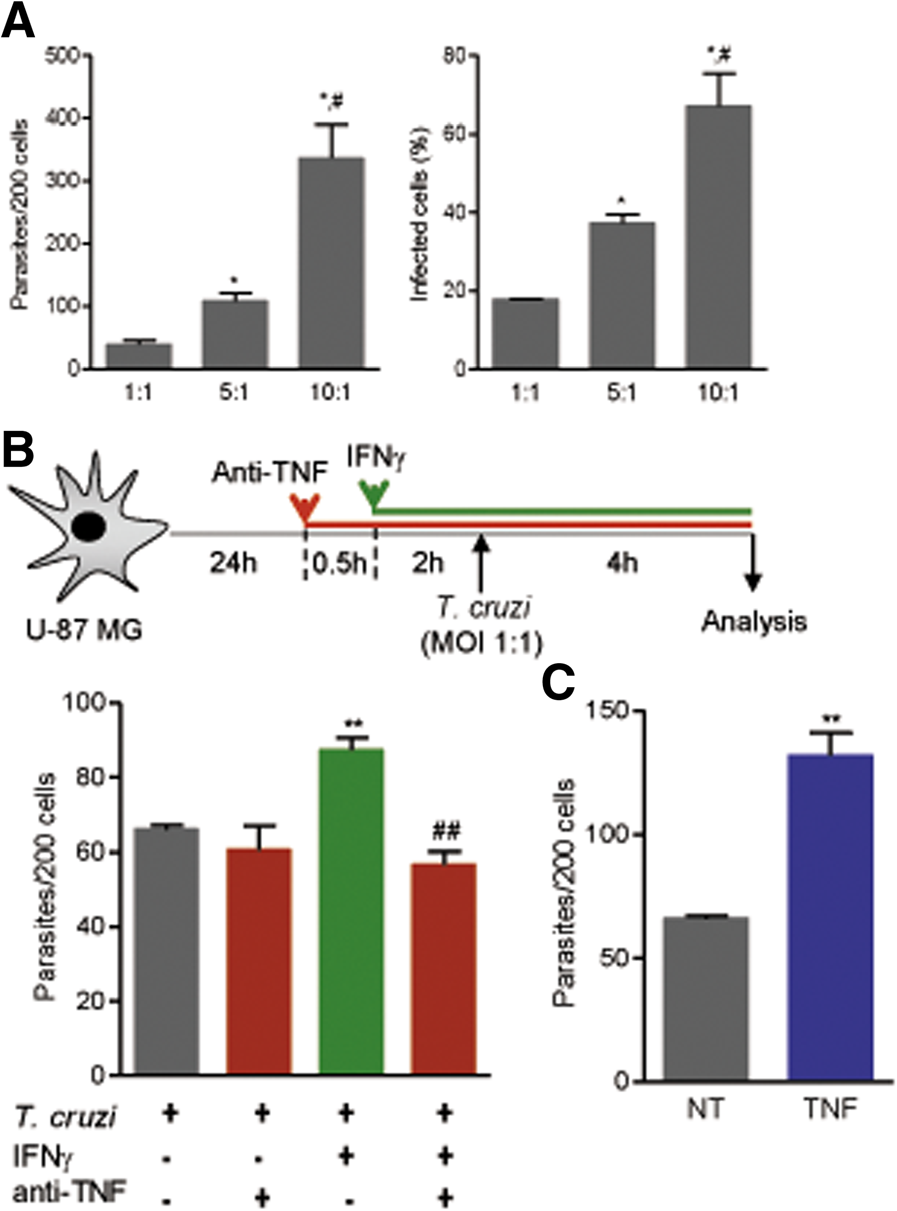
TNF fuels *Trypanosoma cruzi* infection of U-87 MG human glioblastoma cells. **a** Number of parasites per 200 cells and percentage of infected cells at a MOI of 1:1, 1:5 or 1:10 and analysis 4 h after infection. ^*^ *p* < 0.05 MOI of 1:1 vs. MOI of 5:1; ^#^ *p* < 0.05 MOI of 5:1 vs. MOI of 10:1. **b** As indicated in the graph, astrocytes were pre-treated with anti-TNF (10 μg/mL, 30 min prior to rIFNγ addition) and rIFNγ (10 ng/ mL, 2 h prior to infection), infected (MOI 1:1), and analyzed 4 h later. The numbers of parasites per 200 cells are shown. **c** U-87 MG astrocytes were pre-treated with rTNF (1 ng/ mL, 2 h prior infection), infected (MOI 1:1), and analyzed 4 h later. The numbers of parasites per 200 cells are shown. Data are presented as the means ± SEM of triplicates. ^**^ *p* < 0.01 treated (NT) vs. cytokine-treated. ^##^ *p* < 0.01 rIFNγ-treated vs. anti-TNF addition prior to rIFNγ treatment

**Fig. 4:**
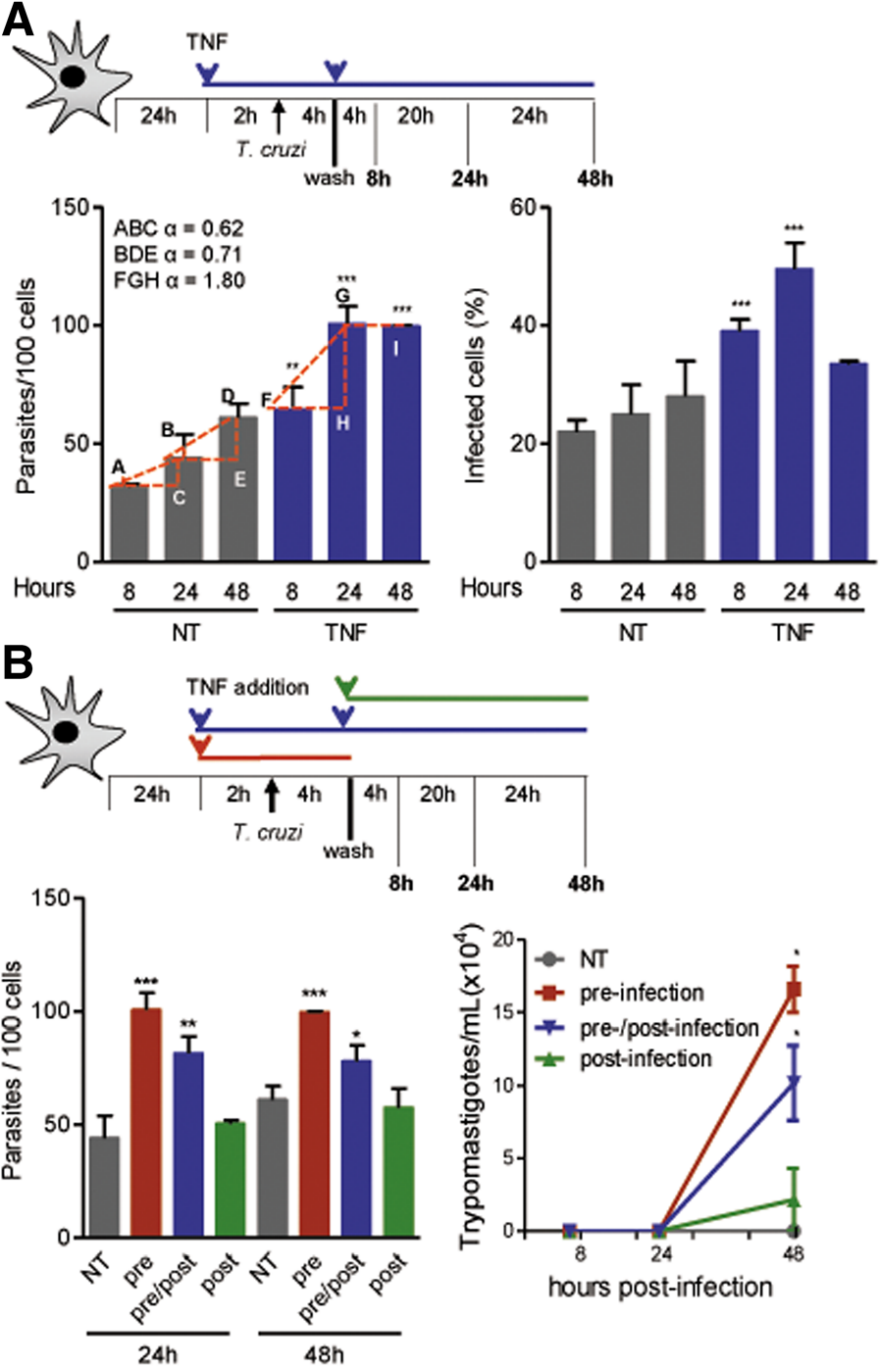
TNF fuels parasite growth and egression of trypomastigote forms from astrocytes. **a** C57BL/6 primary astrocyte cultures were treated with rTNF 2 h before *T. cruzi* infection, and cultures were analyzed after 8, 24, and 48 h of infection. The numbers of parasites per 100 cells and percentage of infected cells are shown. Estimation of the variation rate of parasite growth A-B-C (0.62), B-D-E (0.71), and F-G-H (1.80). **b** Astrocytes were treated with rTNF (1 ng/mL): prior to infection and removed after (pre), prior to and after infection (pre/post), or only after infection (post). The numbers of intracellular forms of the parasite per 100 astrocytes and trypomastigote forms present in the supernatants are shown. Data are presented as the means ± SEM of triplicates. ^*^ *p* < 0.05; ^**^ *p* < 0.01; ^***^ *p* < 0.001 untreated (NT) controls vs. rTNF treatment under the distinct experimental conditions

**Fig. 5:**
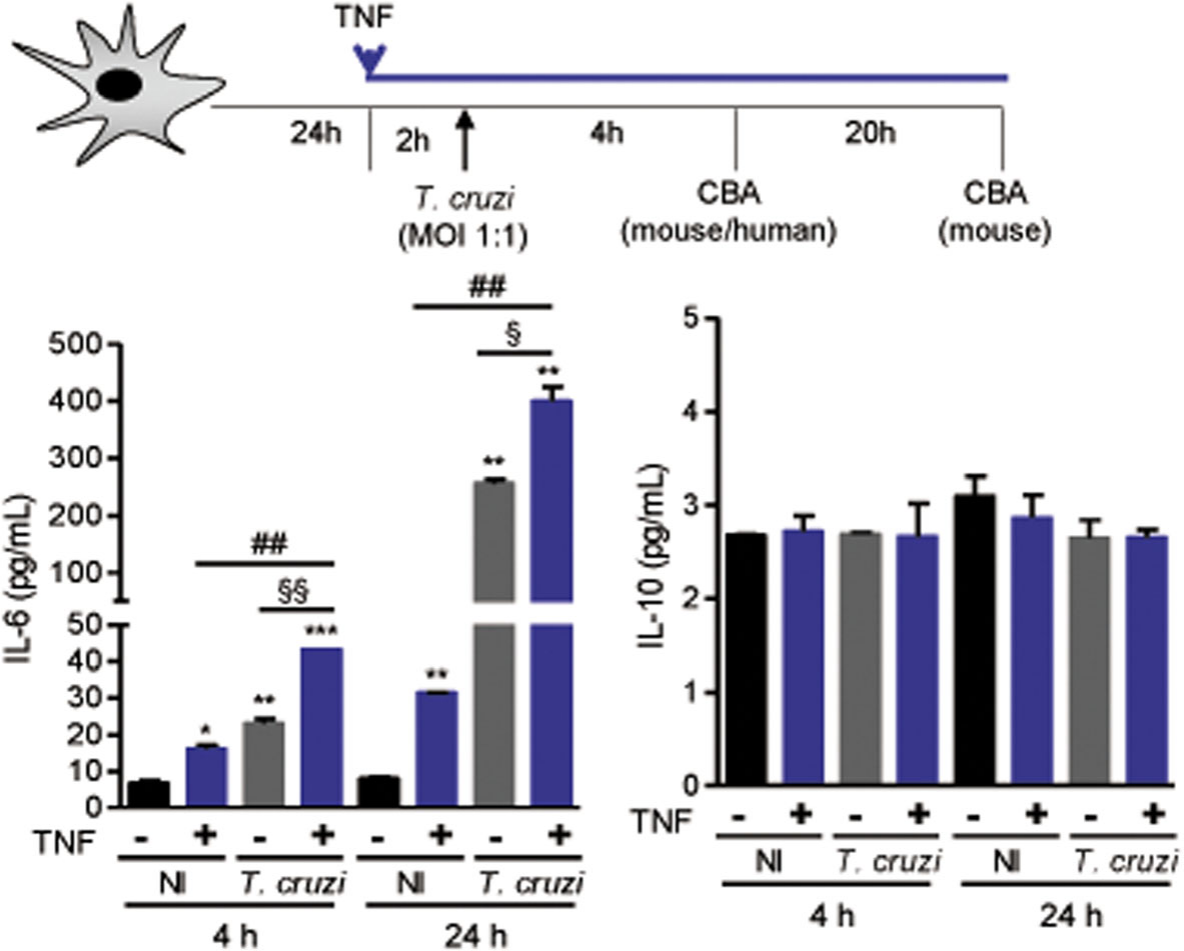
*Trypanosoma cruzi* infection-induced IL-6 production by astrocytes is exacerbated by pre-treatment with TNF. C57BL/6 primary astrocyte cultures were pre-treated with rTNF (1 ng/mL) and infected (MOI 1:1), and then the supernatants were collected after 4 and 24 h of infection and analyzed to detect cytokines by CBA. The concentrations of IL-6 and IL-10 are shown. Data are presented as the means ± SEM of triplicates. ^*^ *p* < 0.05, ^**^ *p* < 0.01, ^***^ *p* < 0.001 untreated (−)/non-infected (NI) vs. other conditions. ^##^ *p* < 0.01 non-infected (NI) vs. *T. cruzi*-infected. ^§^ *p* < 0.05, ^§§^ *p* < 0.01 untreated vs. rTNF pre-treated infected astrocytes

**Fig. 6:**
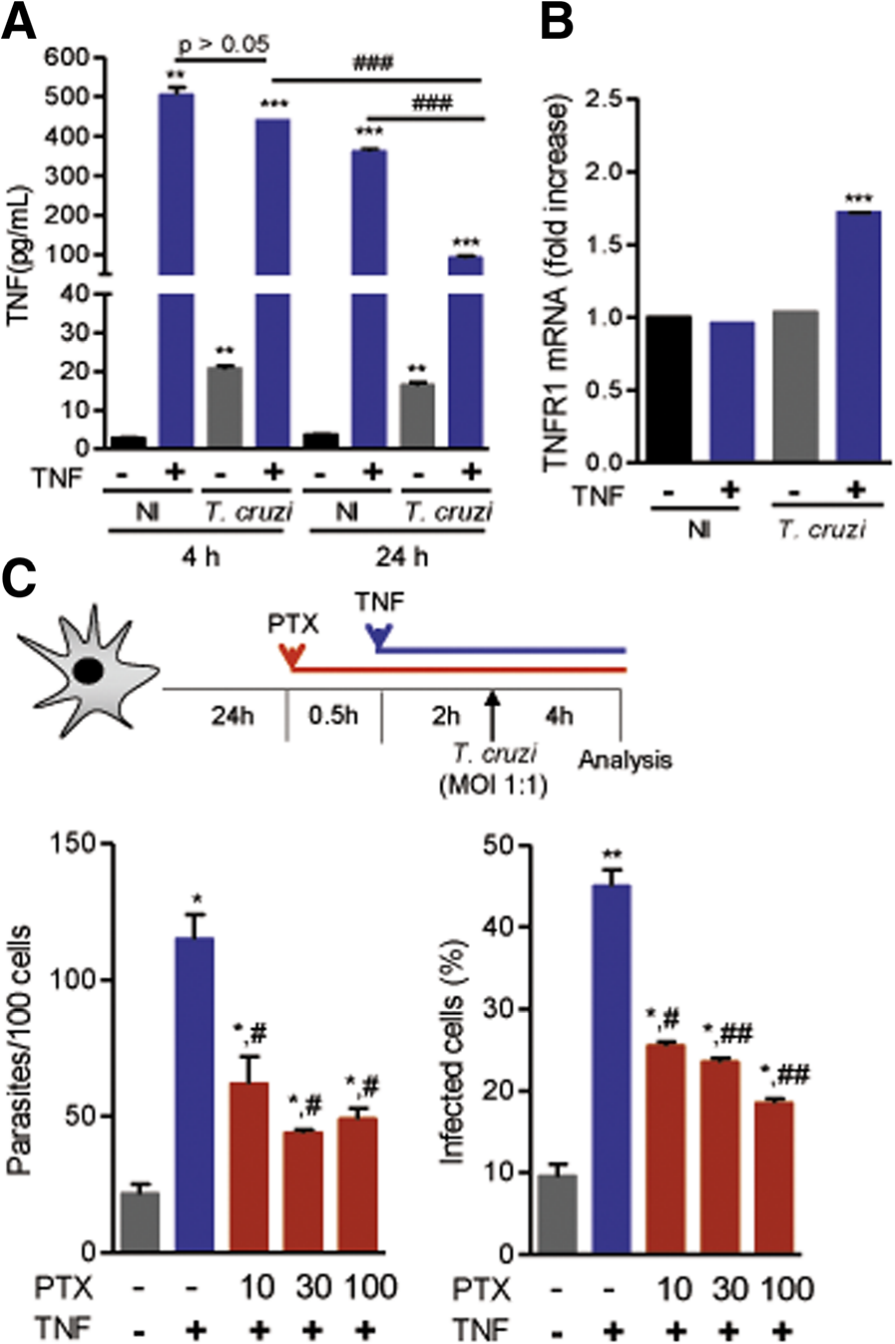
Two signals induce *Tnfr1* mRNA expression, and PTX inhibits rTNF-induced astrocyte invasion by *Trypanosoma cruzi*. **a** Astrocytes were pre-treated with rTNF (1 ng/mL) and infected (MOI 1:1), and supernatants were collected after 4 and 24 h of infection and analyzed for TNF by CBA. **b** *Tnfr1* transcription was detected by RT-qPCR in astrocyte cultures pre-treated with rTNF (1 ng/mL), infected (MOI 1:1) and analyzed 4 h postinfection. ^*^ *p* < 0.05, ^**^ *p* < 0.01, ^***^ *p* < 0.001 untreated (−)/non-infected (NI) vs. other conditions. ^###^ *p* < 0.001 non-infected (NI) vs. *T. cruzi*-infected. **c** Experimental scheme showing that C57BL/6 primary astrocyte cultures were pre-treated with PTX (addition 30 min before rTNF; 10, 30, 100 μg/mL), treated with rTNF (1 ng/mL), and infected with *T. cruzi*. After 4 h of infection, the numbers of parasites per 100 cells and the frequency of infected cells were analyzed. Data are presented as the means ± SEM of triplicates. ^*^ *p* < 0.05 untreated (−/−) vs. other conditions. ^#^ *p* < 0.05, ^##^ *p* < 0.01 rTNF-treated vs. PTX-treated, rTNF-treated infected astrocytes

To investigate the effects of IFNγ and TNF on the *T. cruzi* infection of astrocytes in vitro, astrocytes were plated at a density of 10^5^ cells per 13-mm-diameter poly-ornithine-coated coverslip in 24-well plates (NUNC, Roskilde, Denmark) and allowed to adhere for 24 h. Cultures were then washed three times with warm PBS to remove non-adherent cells, and fresh culture medium supplemented with FBS was added. Cultures of U-87 MG human astrocytes or murine primary astrocytes were left untreated or were pre-treated with human or murine recombinant IFNγ (rIFNγ, 10 ng/mL, eBioscience, San Diego, CA, USA) or TNF (rTNF, 0.1, 0.5, 1, 5, or 10 ng/mL, eBioscience, San Diego, CA, USA) for different time periods depending on the experimental design (Figs. 1, 2, 3, 4, 5, and 6 and Additional file 3: Figure S3, Additional file 4: Figure S4, Additional file 5: Figure S5, Additional file 6: Figure S6, Additional file 7: Figure S7). Infection was performed with trypomastigotes of the Colombian *T. cruzi* strain at the indicated MOI. In a set of experiments, rTNF was added after infection, as shown in the experimental design (Fig. 4). The plates were incubated at 37 °C with 95% humidity and 5% CO_2_. Coverslips were then washed twice with warm PBS, and the cells were fixed in fresh 4% paraformaldehyde for 10 min. After three washes with PBS, the cells were stained with Giemsa, and the coverslips were mounted onto slides with Entellan (Merck, Darmstadt, Germany). The numbers of non-infected and *T. cruzi*-infected astrocytes (300 cells/coverslip) and of intracellular amastigote forms per infected cell were counted. Representative images were acquired on a light microscope coupled with a digital camera DS-L3 (Nikon Corporation, Sendai, Japan).

### Anti-TNF treatment of astrocytes

To evaluate the role of rIFNγ-induced TNF in astrocyte infection, cell cultures were treated with the anti-TNF chimeric monoclonal antibody infliximab (10 μg/mL, Remicade, a gift of Schering-Plough, São Antônio, SP, Brazil) 30 min before the addition of murine or human rIFNγ, as described in the experimental design (Fig. 3).

### Pentoxifylline treatment of astrocytes

Pentoxifylline (PTX), a phosphodiesterase inhibitor, has previously been shown to suppress *Tnfr1* mRNA expression in the experimental model of hepatic ischemia-reperfusion injury [35] and TNFR1 expression in experimental *T. cruzi* infection [36]. To explore the effect of PTX on astrocytes, solutions at a final concentration of 30 μg/mL or 100 μg/mL PTX (Trental, Sanofi, São Paulo, SP, Brazil) were added to astrocytes 30 min prior to rTNF treatment, as described in the experimental design (Fig. 6).

### Astrocyte cell viability

Astrocyte cell viability was observed by the reduction of MTT (3-(4,5-dimethylthiazol-2-yl)-2,5-diphenyltretrazolium bromide; Sigma-Aldrich, Steinheim, Germany). Viable cells convert the yellow, water-soluble tetrazolium salt to insoluble purple formazan. After 5 min, formazan was solubilized with dimethyl sulfoxide (DMSO; Sigma-Aldrich, St. Louis, MO, USA), and the optical density was evaluated at 490 nm using a microplate reader (Asys Expert Plus, Biochrom, Cambridge, UK).

### Cytokine quantification

TNF concentrations in cell culture supernatants and mouse sera were evaluated using an ELISA kit for the detection of mouse TNF (R&D System, Minneapolis, MN, USA) according to the manufacturer’s protocol, and the colorimetric reaction was assessed on an ELISA plate reader at 490 nm (Asys Expert Plus, Biochrom, Cambridge, UK). Cytokines (IL-2, IL-4, IL-6, IL-10, IL-17A, and TNF) were detected in astrocyte culture supernatants using the commercial cytometric bead array (CBA) Mouse Th1/Th2/Th17 Cytokine Kit (# 560485, Becton-Dickinson, San Jose, CA, USA) and Human Inflammatory Cytokines Kit (# 551811, Becton-Dickinson, San Jose, CA, USA) according to the manufacturer’s instructions. The fluorescence produced by the beads was measured on a FACSCalibur flow cytometer (BD, Biosciences, San Jose, CA, USA) and analyzed using the kit’s FCAP Array software. In the ELISA and CBA tests, standard curves (1 pg/mL to 100 ng/mL) were generated in parallel. These methods consistently detected concentrations above 10 pg/mL.

### RT-PCR assay to measure in vivo *Tnf* mRNA expression

*Tnf* mRNA expression in brain tissue was assessed and analyzed as previously described [37]. Briefly, RNA was isolated from mouse brains via acid guanidinium thiocyanate-phenol-chloroform extraction: RNA STAT-60TM. Total RNA (0.5 μg) was reverse transcribed by the addition of 10 U of RNAsin, 15 ρM oligo DT15 (Promega Corp., Madison, WI, USA), and AMV Reverse Transcriptase (RT) (Gibco, Gaithersburg, MD, USA) in 25-μL reactions containing 250 mM dNTPs, 50 mM Tris-HCl, pH 8.3, 75 mM KCl, 3 mM MgCl_2_, and 10 mM DTT. The mixtures were incubated for 5 min at 95 °C, 5 min on ice, and 5 min at 25 °C. At this step, 100 U of RT was added to each sample, and the reaction mixture was incubated for 60 min at 37 °C. The temperature was then elevated to 95 °C for 5 min, and the tubes were transferred to ice for 5 min. The cDNA products were diluted in 0.2 mL of sterile distilled water and used at 5 μL per reaction for PCR amplifications. The PCR was performed in 25-μL reactions with samples diluted in the following buffer: 250 mM dNTPs, 10 mM Tris-HCl, pH 8.3, 50 mM KCl, 1.5 mM MgCl_2_, 10 mM of each primer, and 0.5 U of Taq polymerase (CenBiot, Pelotas, RS, Brazil). After an initial incubation for 3 min at 95 °C, the cycling conditions were denaturation for 1 min at 94 °C, annealing for 1 min at 54 °C, and extension for 2 min at 72 °C. After the designated cycle numbers for each primer, the program executed a final extension of 7 min at 72 °C. The PCR products and molecular weight markers were separated on 6% polyacrylamide gels and stained with silver nitrate. Densitometry was carried out on a CS-9301PC Densitometer (Shimadzu, Tokyo, Japan). The PCRs were standardized using hypoxanthine-guanine phosphoribosyl transferase (HPRT). The same primer sequences and PCR conditions indicated previously were here used for detection of *Tnf* and *Hprt* mRNA [38]. Fold-increases were determined relative to non-infected controls.

### Real-time RT-qPCR to measure in vitro *Tnf* and *Tnfr1* mRNA expression

For real-time quantitative RT-PCR (RT-qPCR), 10^6^ astrocytes were cultivated in a 6-well plate (NUNC, Roskilde, Denmark), infected with *T. cruzi* (MOI 1:1), and cultivated as described above. After 4 h of culture, astrocytes were washed with warm PBS to remove debris, harvested by gentle scraping with a rubber policeman, and frozen in RNAlater (# AM7021, Life Technologies, Carlsbad, CA, USA). Total RNA was extracted using TRI-Reagent (Sigma-Aldrich, St. Louis, MO, USA), after the complete removal of RNAlater. All reverse transcriptase reactions were performed using the SuperScript VILO cDNA Synthesis Kit (Life Technologies, Carlsbad, CA, USA), and real-time RT-qPCR was performed on a Real-Time PCR System (Life Technologies, Carlsbad, CA, USA) using TaqMan gene expression kits (Life Technologies, Carlsbad, CA, USA) for *Tnf* (# Mm00443258_m1) and *Tnfr1* (#Mm00441883_g1), as well as the endogenous housekeeping control genes glyceraldehyde 3-phosphate dehydrogenase (GAPDH) (# Mm99999915_g1) and β actin (# Mm00607939_s1), purchased from Life Technologies (Carlsbad, CA, USA). The reactions were performed and analyzed as described previously [28].

### Experimental in vivo *T. cruzi* infection

**Fig. 7:**
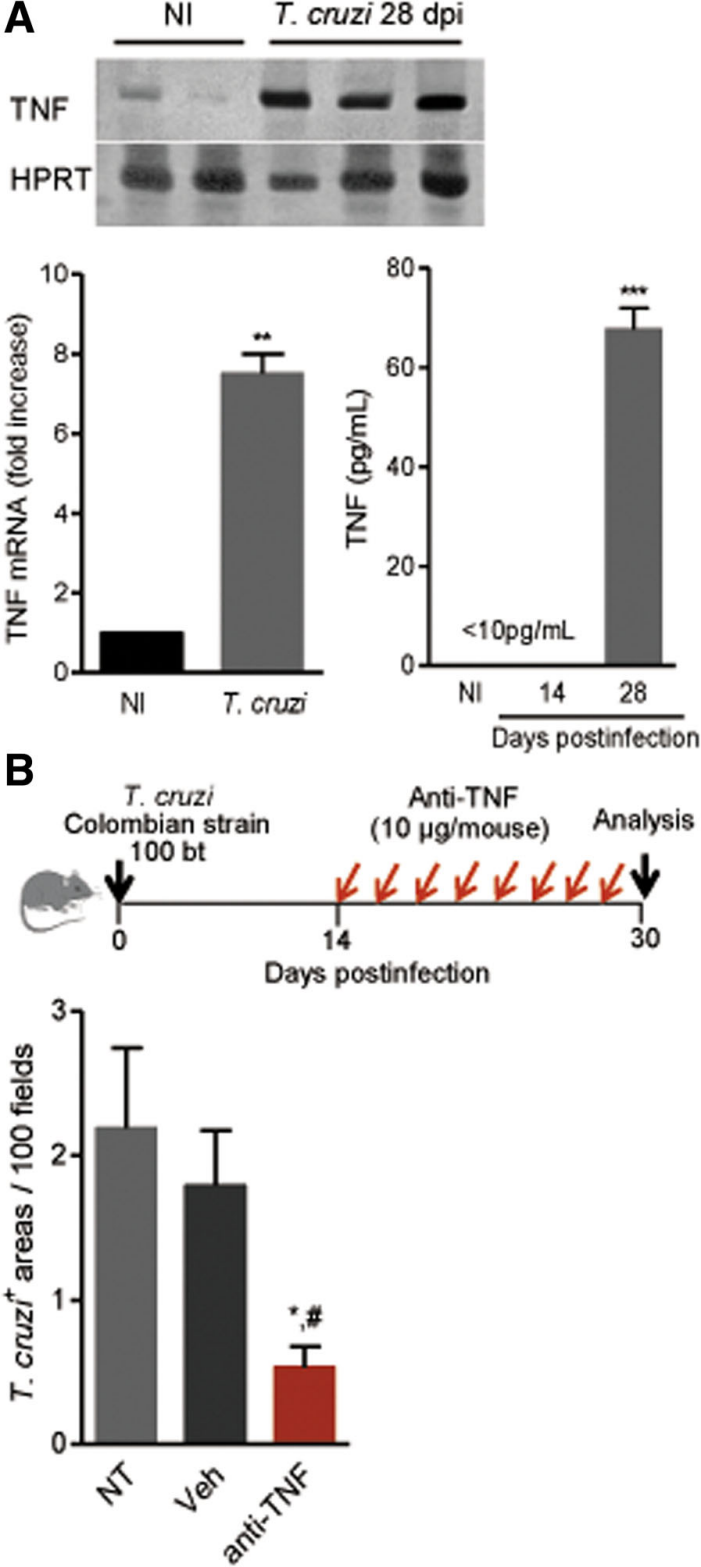
Anti-TNF treatment of *Trypanosoma cruzi*-infected mice reduces the number of parasite nests in the CNS. C3H/He mice were infected with 100 blood trypomastigotes of the Colombian strain. **a** *Tnf* mRNA expression in brain tissue was detected by RT-PCR, and serum TNF concentrations were detected by ELISA. *Hprt* was used as a housekeeping gene. **b** Acutely *T. cruzi*-infected mice were treated at 48-h intervals with the anti-TNF neutralizing antibody infliximab (10 μg/mouse) from 14 to 28 dpi and analyzed at 30 dpi using immunohistochemistry to detect *T. cruzi* antigen-positive areas in the brains of 5–8 animals per group. Data are presented as the means ± SEM of triplicates. ^**^ *p* < 0.01, ^***^ *p* < 0.001 NI vs. *T. cruzi*-infected mice. *Asterisk* untreated (NT) vs. anti-TNF-treated infected mice; ^#^ *p* < 0.05 vehicle-injected (Veh) vs. anti-TNF-treated infected mice

Five- to 7-week-old female C3H/He (H-2^K^) mice obtained from the animal facilities of the Oswaldo Cruz Foundation (CECAL/Fiocruz, Rio de Janeiro, RJ, Brazil) were housed under specific pathogen-free conditions with a 12-h light/dark cycle and ad libitum access to food and water*.* Mice were intraperitoneally infected with 100 blood trypomastigotes of the Colombian Type I *T. cruzi* strain obtained by passages through C3H/He every 5 weeks. This infection model has been shown to develop self-resolving acute meningoencephalitis [25, 27]. The experimental groups were composed of six to ten *T. cruzi*-infected mice and three to five non-infected controls per experiment, in three independent experiments (Fig. 7).

### In vivo anti-TNF treatment

At 14 days postinfection (dpi), when the first parasites were detected in peripheral blood, the mice were subcutaneously treated with injection-grade saline (BioManguinhos-Fiocruz, Rio de Janeiro, RJ, Brazil) containing 10 μg of anti-TNF antibody (Remicade®, Infliximab, Schering-Plough São Antônio, SP, Brazil), at 48-h intervals over 14 days (Fig. 7). Infliximab was previously shown to block TNF activity in vivo in murine models [39] and to decrease serum TNF levels and *Tnf* mRNA expression in the heart tissue in *T. cruzi*-infected mice [40].

### Immunohistochemical staining for parasites

Groups of non-infected and infected mice, with or without anti-TNF treatment, were sacrificed under anesthesia (100 mg/kg ketamine associated with 5 mg/kg xylazine chloride, intraperitoneally), at 30 dpi. The brains were removed, embedded in tissue-freezing medium (Tissue-Tek, Miles Laboratories, Torrance, CA, USA), and stored in liquid nitrogen. Sagittal brain tissue serial cryostat sections (8 μm thick) were fixed in cold acetone and subjected to immunohistochemical staining. Parasite antigens were revealed by staining with polyclonal rabbit anti-*T. cruzi* antibody (produced in our laboratory, LBI/IOC-Fiocruz, Rio de Janeiro, RJ, Brazil) and FITC-labeled goat anti-rabbit secondary antibody (Amersham Life Science, Buckinghamshire, UK). Negative-control brain tissue sections were generated by omitting the primary antibody. Ten sections were analyzed for each studied encephalon. Images were acquired on a fluorescence microscope equipped with a digital camera Eclipse CI (Nikon Corporation, Tokyo, Japan) and analyzed with the Nis-Elements BR 4.0 software (Nikon Corporation, Tokyo, Japan). Areas containing parasites were identified and counted. Data are expressed as *T. cruzi-*positive areas per 100 microscopic fields.

### Statistical analysis

Data are expressed as arithmetic means ± standard error (SE). For statistical analyses, we used Student’s *t* test to compare two groups. Comparisons between groups were carried out by analysis of variance (ANOVA) followed by Bonferroni post hoc test. All statistical tests were performed with GraphPad Prism 5.0 (La Jolla, CA, USA). Differences were considered statistically significant when *p* < 0.05.

## Results

### *T. cruzi*-infected astrocytes produce TNF

The established primary cell cultures of brain cortex astrocytes were composed mainly of GFAP^+^ cells (≥ 99%), while CD11b^+^ cells (< 1%) were rarely detected (Additional file 1: Figure S1) and were therefore enriched in astrocytes, as recommended for the study of monotypic cell cultures [41, 42]. At 4 h of *T. cruzi*/astrocyte interaction using initial MOIs of 1:1 and 10:1, nearly 25 and 60% of astrocytes, respectively, were infected (Additional file 2: Figure S2). At 24 h of *T. cruzi*/astrocyte interaction using MOIs of 1:1 and 10:1, more than 70 and 90% of astrocytes, respectively, harbored amastigote-like forms in the cytoplasm (Additional file 2: Figure S2). Therefore, these monotypic astrocyte cultures obtained from C57BL/6 mice are highly susceptible to *T. cruzi* infection. Next, we investigated whether *T. cruzi*-infected astrocytes express *Tnf* mRNA and produce this cytokine. Compared with non-infected astrocytes, increased *Tnf* mRNA expression (*p* < 0.001) and TNF levels (*p* < 0.001) were detected after 4 h of *T. cruzi*/astrocyte interaction (Fig. 1a). Moreover, compared with non-infected astrocytes (*p* < 0.001) and untreated *T. cruzi*-infected astrocytes (*p* < 0.05), TNF production was upregulated in rIFNγ-treated *T. cruzi*-infected astrocytes (Fig. 1b).

### Pre-treatment of astrocytes with TNF stimulates infection by *T. cruzi*

To examine the influence of TNF on *T. cruzi*/astrocyte interaction and parasite growth inside astrocytes, primary astrocyte cultures were pre-treated with rTNF and then infected with trypomastigote forms at MOIs of 1:1 and 10:1 (Fig. 2a). After 4 h of interaction (MOI of 1:1), the parasite load inside the invaded astrocyte and frequency of infected astrocytes were higher when rTNF was added prior to *T. cruzi* infection (*p* < 0.05) than for untreated astrocytes (Fig. 2b). As similar results were observed when 1 or 10 ng/mL was used, we adopted a final concentration of 1 ng/mL for subsequent experiments. Notably, the addition of rTNF (0.5 to 10 ng/mL) to primary astrocyte cell cultures had no cytotoxic effects on astrocytes (Additional file 3: Figure S3). Compared with the corresponding untreated controls, pre-exposing astrocytes to rTNF increased cell invasion by *T. cruzi* when the initial MOI was 1:1 (*p* < 0.05) or 10:1 (*p* < 0.05) (Fig. 2c). TNF addition prior to infection also increased the frequency of infected astrocytes when the initial MOI was 1:1 (*p* < 0.01), but had no impact when the MOI was 10:1 (*p* = 0.339), when a high frequency (75–80%) of infected cells was already observed in untreated astrocytes (Fig. 2c).

To test whether the ability of TNF to promote *T. cruzi*/astrocyte interaction was a common biological process in other cell types, L-929 mouse fibroblasts were pre-treated with TNF and then infected with *T. cruzi* (Additional file 4: Figure S4A). Pre-exposing fibroblasts to TNF had no effect on the *T. cruzi*/fibroblast interaction (*p* > 0.05), regardless of the MOI (1:1 or 10:1) and time (4 or 24 h) of interaction (Additional file 4: Figure S4B-C).

### Infection of human astrocytes with *T. cruzi* is also fueled by TNF

To investigate the effect of TNF on the infection of human astrocytes by *T. cruzi*, we used the U-87MG astrocyte cell lineage. After 4 h of infection, the numbers of parasites/astrocyte (*p* < 0.05) and frequencies of *T. cruzi*-infected astrocytes (*p* < 0.05) were dependent on the initial MOI (Fig. 3a). Treating U-87MG astrocytes with rIFNγ prior to *T. cruzi* infection increased (*p* < 0.01) the number of amastigote-like forms inside astrocytes (Fig. 3b). Furthermore, the ability of rIFNγ to fuel astrocyte infection was prevented (*p* < 0.01) by the addition of the anti-TNF antibody infliximab prior to rIFNγ treatment (Fig. 3b). Conversely, the addition of human rTNF prior to infection increased (*p* < 0.01) the mean number of parasites inside U-87MG astrocytes compared with that of untreated astrocytes (Fig. 3c).

### TNF increases parasite growth inside astrocytes

To analyze the effect of TNF on *T. cruzi* growth inside astrocytes, we performed a kinetic study. Astrocytes from C57BL/6 mice were seeded for 24 h and then either left untreated or were treated with rTNF, submitted to infection, washed to remove non-internalized trypomastigotes, and cultured for 8, 24, and 48 h (Fig. 4a). At these time intervals, compared with the corresponding untreated controls, an increased number of intracellular forms was detected in rTNF-treated astrocytes (*p* < 0.01, 8 h; *p* < 0.001, 24 and 48 h). Comparing the mean number of parasites inside astrocytes at 8 and 24 h of interaction revealed that the proliferation ratio of amastigote forms inside rTNF-treated astrocytes (α = 1.8) was almost threefold higher than that inside untreated astrocytes (*α* = 0.62). At 8 and 24 h of infection, compared with their respective untreated control astrocytes, the frequencies of infected astrocytes were also augmented in rTNF-treated astrocytes (*p* < 0.001). At 48 h of infection, the frequency of infected astrocytes was drastically reduced when cells were pre-treated with rTNF, suggesting the rupture of astrocytes harboring a high number of parasites (Fig. 4a).

### TNF favors parasite egression from astrocytes

Initially, we tested whether the effect of rTNF on astrocyte infection by *T. cruzi* depends on the timing of cytokine exposure: prior to infection, prior to and sustained during infection, or after infection (Fig. 4b). At 24 and 48 h postinfection, the mean number of parasites per astrocyte was increased when rTNF was added to target cells prior to and removed after infection compared with that in the corresponding untreated control astrocytes (*p* < 0.001, for both 24 and 48 h). At these time-points, higher parasite loads were also detected when rTNF was added prior to and sustained after infection than for the corresponding controls (*p* = 0.01, 24 h; *p* < 0.05, 48 h). However, when rTNF was added after *T. cruzi* infection, no effect (*p* = 0.586, 24 h; *p* = 0.206, 48 h) on the parasite load was seen compared with the corresponding untreated astrocytes (Fig. 4b). Similarly, we evaluated the frequency of infected cells stratified into classes based on the number of parasites within their cytoplasm. At 24 h of interaction, compared with the corresponding untreated control astrocytes, the addition of rTNF in the three experimental conditions enhanced (*p* < 0.05) the frequency of astrocytes harboring a higher number of amastigote-like forms. At 48 h of interaction, compared with the corresponding untreated control astrocytes, both experimental conditions using rTNF prior to infection enhanced the frequency of astrocytes harboring > 6 amastigotes/astrocyte (*p* < 0.05); however, no change was detected when rTNF was added after infection (Additional file 5: Figure S5). Together, these data suggest that in addition to fueling parasite/astrocyte interaction, pre-treating astrocytes with rTNF prior to *T. cruzi* infection may also favor parasite egression. Therefore, to test this idea, astrocytes were submitted to rTNF treatment at different time-points as described above, and the number of trypomastigotes in the supernatant was counted at 8, 24, and 48 h of infection. Trypomastigotes emerged from untreated astrocytes after 96 or 120 h (data not shown). At 8 and 24 h of infection, no trypomastigotes were detected in any of the cell culture supernatants. At 48 h of infection, compared with the corresponding untreated control astrocytes, a significant increase in the egression of trypomastigote forms was detected when rTNF was added prior to and removed after infection or was added prior to and sustained after infection (*p* < 0.05, for both conditions), but no significant effect (*p* = 0.431) was detected when rTNF was added after infection (Fig. 4b).

### rTNF amplifies the pro-inflammatory profile of astrocytes exposed to *T. cruzi* infection

The effect of TNF on cytokine production in the presence of *T. cruzi* infection was analyzed using CBA assays to detect mouse and human Th1/Th2/Th17 cytokines (Fig. 5; Additional file 6: Figure S6). At 4 and 24 h, increased concentrations of IL-6 were detected in the supernatants of *T. cruzi*-infected mouse astrocyte cultures (*p* < 0.01, for both time-points) compared with non-infected astrocytes. Treating non-infected astrocytes with rTNF also enhanced (*p* < 0.05, 4 h; *p* < 0.01, 24 h) IL-6 concentrations in the culture supernatants (Fig. 5). Furthermore, at both time-points, rTNF exposure prior to *T. cruzi* infection of mouse astrocytes increased IL-6 production compared with untreated and non-infected (*p* < 0.001, 4 h; *p* < 0.01, 24 h), rTNF-treated non-infected (*p* < 0.01, both time-points), and *T. cruzi*-infected astrocytes (*p* < 0.01, 4 h; *p* < 0.05, 24 h), although a synergistic effect of rTNF and *T. cruzi* infection was not detected. By contrast, under all experimental conditions, the regulatory cytokine IL-10 was barely detected and was comparable to non-infected controls (Fig. 5). An inflammatory milieu with increased concentrations of IL-6 was also detected in the supernatants of *T. cruzi*-infected (*p* < 0.05), rTNF-treated (*p* < 0.05), or rTNF-primed and infected (*p* = 0.063) human U-87MG astrocytes. Under these conditions, IL-10 was not detected (Additional file 6: Figure S6). At 4 and 24 h, *T. cruzi*-infected mouse astrocytes released higher levels of TNF than non-infected astrocytes (*p* < 0.05, both time-points). Interestingly, a significant decay (*p* < 0.001) in TNF concentrations in the supernatants of rTNF-treated *T. cruzi*-infected astrocytes compared with rTNF-treated non-infected astrocytes was observed at 24 h, suggesting consumption of the added TNF (Fig. 6a).

### Two signals promptly induce *Tnfr1* expression

The apparent consumption of the added rTNF by *T. cruzi*-infected astrocytes (Fig. 6a) led us to wonder whether TNF receptors were induced by rTNF or *T. cruzi* infection. We therefore analyzed *Tnfr1* mRNA expression by RT-qPCR. At 4 h after infection, similar *Tnfr1* transcript levels were detected in untreated or rTNF-treated non-infected astrocytes and in *T. cruzi*-infected astrocytes. Importantly, *T. cruzi* infection of rTNF-primed astrocytes promptly increased (*p* < 0.001) *Tnfr1* mRNA expression (Fig. 6b), supporting the hypothesis that these two signals are required to upregulate TNFR1 expression on astrocytes.

### Pentoxifylline inhibits TNF-fueled astrocyte infection by *T. cruzi*

PTX, a methylxanthine derivative and nonspecific phosphodiesterase inhibitor, diminishes TNFR1 expression in non-glial cells [35, 36]. Thus, we examined whether PTX would interfere with TNF-fueled *T. cruzi*/astrocyte interaction. To test this idea, astrocytes were sequentially pre-treated with PTX, treated with rTNF, and submitted to *T. cruzi* interaction (Fig. 6c). The rTNF-induced increase in the number of amastigote-like forms/astrocyte was partially abrogated by PTX addition at 10, 30, or 100 μg/mL (*p* < 0.05, for all three concentrations). PTX treatment also partially inhibited (*p* < 0.05, 10 μg/mL; *p* < 0.01, 30 and 100 μg/mL) the rTNF-fueled augmentation of the frequency of *T. cruzi*-infected astrocytes (Fig. 6c).

### In vivo *T. cruzi* infection of C3H/He mice enhances TNF expression in the CNS and systemically

Acutely *T. cruzi*-infected C3H/He mice present meningoencephalitis in the presence of brain parasitism [25, 29]. Hence, we examined the impact of *T. cruzi* infection on TNF expression in the CNS and systemically in acutely (14 and 28 dpi) Colombian-infected C3H/He mice. At 28 dpi, increased brain *Tnf* mRNA expression (*p* < 0.01) and high serum TNF concentrations (*p* < 0.001) were detected in infected mice compared with those in non-infected controls (Fig. 7a).

### Anti-TNF diminishes parasite load in the CNS of acutely *T. cruzi*-infected C3H/He mice

Although not detected at 7 and 14 dpi (data not shown), at 30 dpi, *T. cruzi* parasite nests were observed in the CNS of Colombian-infected C3H/He mice (Fig. 7b). Initially, we found that the ability of rTNF to fuel the infection of primary C57BL/6 astrocyte cultures by *T. cruzi* was conserved in astrocytes from the C3H/He mouse lineage (Additional file 7: Figure S7). Next, we tested whether TNF fuels in vivo *T. cruzi* parasitism in the CNS using infected C3H/He mice treated at 48-h intervals with the anti-TNF antibody infliximab from 14 to 28 dpi and analyzed at 30 dpi. Notably, the number of *T. cruzi*-positive areas in the brain was significantly reduced in anti-TNF-treated mice compared with untreated or vehicle-injected (*p* < 0.05, for both conditions) acutely infected C3H/He mice (Fig. 7b).

## Discussion

In the present study, we show that (i) TNF is produced by *T. cruzi*-infected astrocytes and is upregulated by IFNγ stimulation prior to infection; (ii) TNF priming fuels astrocyte infection, accelerates amastigote multiplication and trypomastigote egression; (iii) TNF-primed infected astrocytes create a TNF- and IL-6-enriched inflammatory milieu; and (iv) TNF/TNFR1 signaling may fuel parasite infection. Furthermore, treating infected mice with TNF antibody revealed that TNF facilitates *T. cruzi* parasitism in the CNS, reinforcing the biological relevance of our in vitro findings.

We first showed that primary astrocyte cultures from C57BL/6 mice and the human U-87 MG lineage are targets of *T. cruzi* infection, corroborating previous data using astrocytes from C3H/He mice [29] and the human CRl-1718 astrocyte lineage [43]. Therefore, susceptibility of astrocytes to *T. cruzi* infection in vitro is preserved in different mouse genetic backgrounds and among species. In Chagas disease patients and experimental models, astrocytes are preferred targets for *T. cruzi* invasion and proliferation in the CNS, regardless of the host species and parasite strain [21, 26, 29, 44]. Thus, the experimental models proposed here are appropriate to test our hypotheses.

Mouse and human astrocytes respond to *T. cruzi* infection by producing TNF, which is upregulated by rIFN stimulation prior to *T. cruzi* infection. In experimental African trypanosomiasis (sleeping sickness), *Tnf* transcripts were detected concomitant with astrocyte activation, indicating that these glial cells may be sources of TNF [45]. Additionally, in a cerebral malaria model, *Tnf* mRNA was detected by in situ PCR in astrocytes [46], indicating that astrocytes are activated and respond by upregulating TNF production in different parasitic diseases. However, the biological consequences of TNF production by astrocytes under these conditions were not entirely explored. In Chagas disease patients, high TNF plasma levels are associated with disease severity [17, 18, 47]. In chronically infected mice, serum TNF concentrations are associated with overall disease severity, including parasitemia and parasite load in the heart tissue [19, 48]. Therefore, we assessed the role of TNF in the *T. cruzi*/astrocyte interaction. Interestingly, the anti-TNF blocking antibody infliximab hampered the IFNγ-induced increase in human astrocyte susceptibility to *T. cruzi* infection, supporting the role of TNF in this process. Indeed, pre-treating murine and human astrocytes with TNF fuels *T. cruzi* invasion, revealing a conserved biological activity of TNF. However, this biological process is not common to all cell types because pre-treating fibroblasts with TNF did not facilitate *T. cruzi* infection.

The ability of TNF to fuel astrocyte invasion by *T. cruzi* depends on when astrocytes experience this cytokine. In fact, this outcome was restricted to treatment prior to infection, but not when TNF was added after infection. Interestingly, treating *T. cruzi*-infected astrocytes with TNF had no effect on parasite growth control. Previously, TNF has been shown to facilitate the invasion of non-professional phagocytic epithelial cell lines by *T. cruzi* via NF-kB activation [49]. Interestingly, in vivo TNF therapy aggravates acute infection [50]. Conversely, TNF improves the control of amastigote multiplication in mouse peritoneal macrophages [50, 51]. Regarding the role of cytokines in *T. cruzi* infection, transforming growth factor β has been shown to enhance *T. cruzi* invasion and replication in epithelial cells [52] and primary cardiomyocyte cultures [53]. However, the influence of cytokines on the ability of *T. cruzi* to complete the cycle allowing egression of trypomastigote forms has not been previously studied. Our data showed that TNF increases intracellular parasite growth and accelerates trypomastigote egression when added to astrocytes prior to infection but not after infection. Therefore, depending on the timing of exposure, TNF favors the complete *T. cruzi* cycle in the primary CNS-resident cell. The possibility that TNF acts on the expression of cell surface molecules or other inflammatory mediators that drive the invasion of and egression from astrocytes by *T. cruzi* cannot be excluded. Indeed, we recently showed that nitric oxide may also contribute to IFNγ-driven astrocyte invasion by *T. cruzi* [29].

Astrocytes infected by *Toxoplasma gondii* create a regulatory milieu with high IL-10 concentrations that downmodulate the inflammatory profile of IFNγ-activated microglia and impair neuronal death [33]. By contrast, high concentrations of TNF and IL-6, but not of the regulatory cytokine IL-10, were produced by astrocytes after *T. cruzi* infection and were upregulated by TNF stimulation. Therefore, in the presence of *T. cruzi* infection, astrocytes likely generate an inflammatory milieu in the CNS. Nevertheless, whether the cytokines released by CNS-resident cells in response to *T. cruzi* infection are beneficial or detrimental remains unclear. In vivo, the production of pro-inflammatory cytokines such as IL-6 and TNF by CNS-resident cells may help clear debris, repair and regenerate nervous tissue. However, prolonged exposure to high levels of these cytokines may worsen or accelerate tissue dysfunction and degenerative processes [12]. IL-6 is a cytokine with pleiotropic effects [54]. In experimental autoimmune encephalomyelitis, astrocyte-derived IL-6 present in neuroinflammatory lesions has been implicated in motor disability and disease progression [55]. In chagasic infection, IL-6 produced in situ may synergize with other cytokines to play a detrimental role in the CNS, a question that should be pursued. Interestingly, *T. cruzi*-infected mice present systemically elevated levels of IFNγ and TNF [19, 28, 40, 47] and IFNγ^+^ Iba-1^+^ glial cells surrounding GFAP^+^ *T. cruzi*-bearing astrocytes [29]. In chronic *T. cruzi* infection, systemic TNF has emerged as a key cytokine in the pathogenesis of cardiomyopathy and the dysregulation of the immune response in the spleen and bone marrow [40]. A collection of data suggest that increased systemic IFNγ- and TNF-induced activation of the tryptophan-degrading enzyme indoleamine 2,3-dioxygenase, as well as neuroinflammation, contribute to behavioral abnormalities [56]. In *T. cruzi* infection, systemically disturbed IFNγ and TNF production may create a TNF-enriched environment in the CNS that may fuel parasitism but also create a deleterious circuit, leading to behavioral alterations. In line with this idea, systemic treatment with anti-TNF antibody or with the TNFR1 modulator PTX ameliorated depressive-like behavior in chronically *T. cruzi*-infected mice [28], suggesting that TNF signaling via TNFR1 may play a key role in behavioral alterations in *T. cruzi* infection.

To shed light on the mechanism by which TNF fuels astrocyte infection by *T. cruzi*, we analyzed TNFR1 expression on these glial cells. TNF failed to upregulate *Tnfr1* transcription, corroborating previous data [10]. However, increased *Tnfr1* mRNA expression was detected in the presence of both TNF and *T. cruzi* infection, accompanied by increased consumption of TNF. Thus, astrocytes may facilitate *T. cruzi* persistence in the CNS by inducing TNF production and, in conjunction, these signals may upregulate TNFR1 expression. Indeed, pre-treating astrocytes with PTX reduced the TNF-induced augmentation of astrocyte invasion by *T. cruzi*. PTX has previously been shown to reduce TNF [57–59] and TNFR1 [35, 36] expression, putatively damping TNF/TNFR1 signaling. Together, these results suggest that *T. cruzi* infection in astrocytes triggers TNF production, which upregulates TNF and TNFR1 expression, creating a feedback circuit that drives the inflammatory milieu and contributes to parasite persistence in the CNS.

Intense CNS parasitism and inflammation by blood-borne leukocytes are detected in the acute phase of *T. cruzi* infection in C3H/He mice [25, 27]. Here, we detected increased brain *Tnf* mRNA expression and high serum TNF concentrations in acutely Colombian-infected C3H/He mice. Therefore, we tested the idea that in vivo TNF fuels *T. cruzi* parasitism in the CNS. Indeed, anti-TNF antibody treatment reduced the number of *T. cruzi*-positive areas in the brain. In chronically *T. cruzi*-infected mice, anti-TNF antibody treatment downregulated systemic TNF and TNFR1 expression [40] and abrogated depressive-like behavior [28]. Interestingly, recent data have shown that acutely high systemic TNF concentrations upregulate *Tnf* transcription in the CNS and produce cognitive dysfunction [60]. Nevertheless, our data suggest that systemic or intrinsically produced TNF may facilitate the entry and housing of parasites into CNS-resident cells in acutely *T. cruzi*-infected mice. High systemic TNF concentrations are also detected in chronically *T. cruzi*-infected mice [19, 40]. Thus, systemic TNF draining from the CNS parenchyma and TNF produced by CNS-resident astrocytes may self-sustain the intrinsic inflammatory milieu and contribute to parasite persistence in the CNS during chronic infection. Although we cannot distinguish what happens first in vivo, increased TNF fueling parasite invasion of astrocytes or parasitism inducing TNF upregulation by CNS-resident cells, we believe that these processes form a feedback loop, which may contribute to parasite persistence in the CNS. Although not explored in the present work, it is tempting to extrapolate our findings to propose that parasite persistence in an apparently silent way concurs to create a potent glial cell-borne inflammatory milieu, which may trigger behavioral alterations in *T. cruzi*-infected individuals.

### Limitations

In vitro models are reductionist approaches and are therefore limited in their ability to reproduce the complexity of the network of interactions occurring in the CNS. Although our in vivo result showing reduced CNS *T. cruzi* parasitism after anti-TNF treatment allowed an approximation with in vitro data, supporting a role for TNF in astrocyte invasion by *T. cruzi*, directly translating our findings to the pathophysiology of the CNS commitment in Chagas disease is undoubtedly the crucial limitation of our study.

## Conclusions

The TNF-enriched inflammatory milieu created by *T. cruzi* infection of astrocytes may fuel parasite infection of these glial cells via TNF/TNFR1 signaling, thereby perpetuating the parasite cycle in the CNS.

## Additional files

Additional File 1: Figure S1. Primary cell cultures of C57BL/6 cerebral cortex are enriched in astrocytes. CNS cells of seven to ten days of primary culture were seeded at a density of 10^5^ cells per 13-mm-diameter coverslip and analyzed 24 h later. (a, b, c) Giemsa staining reveals cells resembling astrocytes with thin extensions, delicate branches, recognizable nucleoli and cytoplasm lightly stained. (d, e, f) Simultaneous immunohistochemical staining for GFAP and CD11b was used to determine the cell composition of primary astrocyte cultures. (e) Insert showing RAW 264.7 mouse macrophage lineage used as positive control for CD11b staining. (TIFF 1266 kb)

Additional File 2: Figure S2. Astrocytes from neonatal C57BL/6 mice are susceptible to in vitro infection by *Trypanosoma cruzi*. (a) Monotypic astrocyte cell cultures were allowed to interact during 4 or 24 h with Vero cells-born trypomastigote forms of the Colombian *T. cruzi* strain, at a MOI of 1:1 or 10:1. (b-e) Astrocyte infected with *T. cruzi* at a MOI of 1:1 and analyzed after 4 h (b, c, d) or 24 h (e) of interaction. (f) Parasites per 100 cells and frequency of *T. cruzi*-infected astrocytes after 4 and 24 h of *T. cruzi*/astrocyte interaction at a MOI of 1:1 or 10:1 Data are presented as means ± SEM of triplicates. ^*^, *p* < 0.05 and ^**^, *p* < 0.01 MOI 1:1 vs. MOI 10:1 in each analyzed interval; ^#^, *p* < 0.05 and ^##^, *p* < 0.01 4 h vs. 24 h of infection in each analyzed MOI. (TIFF 1349 kb)

Additional File 3: Figure S3. Addition of rTNF to astrocyte cultures had no cytotoxic effects on astrocytes. C57BL/6 primary astrocyte cultures were seeded at a density of 10^5^ cells per 13-mm-diameter coverslip, 24 h later washed with warm PBS and left untreated (NT), treated with rTNF (0.5, 1, 5, 10 ng/mL) or toxic agents (HCl pH 4.0 or DMSO). After 24 h of exposure, cells were submitted to MTT assay. Data are presented as means ± SEM of triplicates. ^***^, *p* < 0.001 untreated (NT) vs. astrocytes submitted to other experimental conditions. (TIFF 881 kb)

Additional File 4: Figure S4. Pre-treatment of fibroblast with rTNF does not affect infection by *Trypanosoma cruzi*. (a) Experimental scheme showing that L-929 fibroblasts were pre-treated with TNF (1 ng/mL) and subsequently infected with trypomastigote forms of the Colombian *T. cruzi* strain. (b-c) The percentage of infected cells and the number of parasites per cells were analyzed at 4 h (b) and 24 h (c) of infection, at a MOI of 1:1 or 10:1. Data are presented as mean ± SEM of duplicates. (TIFF 1430 kb)

Additional File 5: Figure S5. Pre-exposing astrocyte cultures to rTNF enhances parasite load in *Trypanosoma cruzi*-infected astrocytes. C57BL/6 primary astrocyte cultures were seeded, exposed to rTNF and infected as described in Fig. 5a. At 24 and 48 h of infection, the frequencies of infected astrocytes were stratified by classes according to the number of amastigote-like forms harbored in the cytoplasm. Data are presented as means ± SEM of triplicates. ^*^, *p* < 0.05, ^**^, *p* < 0.01 untreated (NT) vs. rTNF-treated astrocytes. (TIFF 1045 kb)

Additional File 6: Figure S6. rTNF amplifies the pro-inflammatory profile of human astrocytes exposed to *Trypanosoma cruzi* infection. Human astrocytes were submitted to treatment with rTNF and left non-infected (NI) or infected by *T. cruzi* (MOI 1:1). At 4 h of infection, supernatants were collected and submitted to detection of cytokines by CBA. Data are presented as means ± SEM of triplicates. ^*^, *p* < 0.05, untreated (NT) vs. rTNF-treated astrocytes. (TIFF 1375 kb)

Additional File 7: Figure S7. rTNF effects on *Trypanosoma cruzi*/astrocyte interaction are conserved in primary astrocyte cultures of C3H/He mice. (a) Monotypic primary astrocyte cell cultures of C3H/He mice were seeded, left untreated or treated with rTNF and infected with trypomastigote forms of the Colombian *T. cruzi* strain at a MOI of 1:1 or 10:1. (b) After 4 h of interaction, the frequencies of infected cells and the number in untreated (NT), rTNF-treated astrocytes are shown. (c) Data show the frequencies of infected astrocytes stratified by classes according to the number of amastigote-like forms harbored in the cytoplasm. Data are presented as means ± SEM of triplicates. ^*^, *p* < 0.05, ^***^, *p* < 0.001 untreated (NT) vs. rTNF-treated astrocytes. ^#^, *p* < 0.05, ^##^, *p* < 0.01, ^###^, *p* < 0.001 MOI 1:1 vs. MOI 10:1. (TIFF 1337 kb)

## Abbreviations

CBA: Cytometric bad array
CD: Cluster of differentiation
CEUA: Committee for Animal Ethics
CNS: Central nervous system
DAPI: 4′,6-Diamidino-2-phenylindole
DMSO: Dimethyl sulfoxide
DNTP: 2′-Deoxynucleoside 5′-triphosphates
DPI: Days postinfection
ELISA: Enzyme-linked immunosorbent assay
FBS: Fetal bovine serum
FITC: Fluorescein isothiocyanate
GFAP: Glial fibrillary acidic protein
HIV: Human immunodeficiency virus
HPRT: Hypoxanthine-guanine phosphoribosyl transferase
Iba-1: Ionized calcium-binding adapter molecule 1
IFN: Interferon
IL: Interleukin
IOC: Instituto Oswaldo Cruz/Oswaldo Cruz Institute
MOI: Multiplicity of infection
MTT: 3-(4,5-dimethylthiazol-2-yl)-2,5-diphenyltetrazolium bromide
NF-kB: Nuclear factor kappa B
NI: Non-infected
NT: Not-treated
PCR: Polymerase chain reaction
PTX: Pentoxifylline
rIFN: Recombinant interferon
rTNF: Recombinant tumor necrosis factor
RT-qPCR: Quantitative real-time polymerase chain reaction
Th: T helper cells
TNF: Tumor necrosis factor
TNFR1: Tumor necrosis factor receptor 1
TNFR2: Tumor necrosis factor receptor 2
