# Supplementary figures and images for: Priming astrocytes with TNF enhances their susceptibility to Trypanosoma cruzi infection and creates a self-sustaining inflammatory milieu
Source: J Neuroinflammation. 2017 Sep 6;14:182. doi: 10.1186/s12974-017-0952-0 (PMC5588596; doi:10.1186/s12974-017-0952-0)

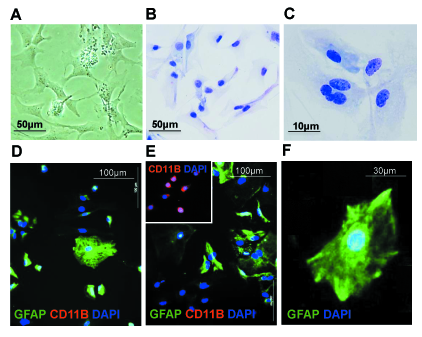

Supplement: Supplementary file 1 — Primary cell cultures of C57BL/6 cerebral cortex are enriched in astrocytes. CNS cells of seven to ten days of primary culture were seeded at a density of 105 cells per 13-mm-diameter coverslip and analyzed 24 h later. (a, b, c) Giemsa staining reveals cells resembling astrocytes with thin extensions, delicate branches, recognizable nucleoli and cytoplasm lightly stained. (d, e, f) Simultaneous immunohistochemical staining for GFAP and CD11b was used to determine the cell composition of primary astrocyte cultures. (e) Insert showing RAW 264.7 mouse macrophage lineage used as positive control for CD11b staining. (TIFF 1266 kb) [file 12974_2017_952_MOESM1_ESM.tif]

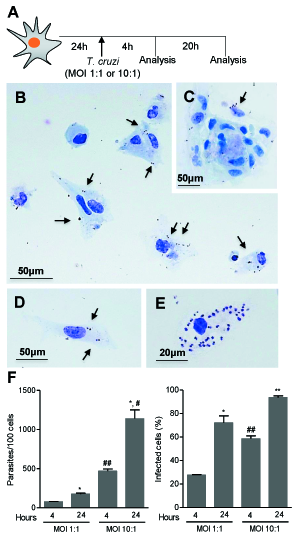

Supplement: Supplementary file 2 — Astrocytes from neonatal C57BL/6 mice are susceptible to in vitro infection by Trypanosoma cruzi. (a) Monotypic astrocyte cell cultures were allowed to interact during 4 or 24 h with Vero cells-born trypomastigote forms of the Colombian T. cruzi strain, at a MOI of 1:1 or 10:1. (b-e) Astrocyte infected with T. cruzi at a MOI of 1:1 and analyzed after 4 h (b, c, d) or 24 h (e) of interaction. (f) Parasites per 100 cells and frequency of T. cruzi-infected astrocytes after 4 and 24 h of T. cruzi/astrocyte interaction at a MOI of 1:1 or 10:1 Data are presented as means ± SEM of triplicates. *, p < 0.05 and **, p < 0.01 MOI 1:1 vs. MOI 10:1 in each analyzed interval; #, p < 0.05 and ##, p < 0.01 4 h vs. 24 h of infection in each analyzed MOI. (TIFF 1349 kb) [file 12974_2017_952_MOESM2_ESM.tif]

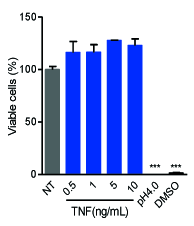

Supplement: Supplementary file 3 — Addition of rTNF to astrocyte cultures had no cytotoxic effects on astrocytes. C57BL/6 primary astrocyte cultures were seeded at a density of 105 cells per 13-mm-diameter coverslip, 24 h later washed with warm PBS and left untreated (NT), treated with rTNF (0.5, 1, 5, 10 ng/mL) or toxic agents (HCl pH 4.0 or DMSO). After 24 h of exposure, cells were submitted to MTT assay. Data are presented as means ± SEM of triplicates. ***, p < 0.001 untreated (NT) vs. astrocytes submitted to other experimental conditions. (TIFF 881 kb) [file 12974_2017_952_MOESM3_ESM.tif]

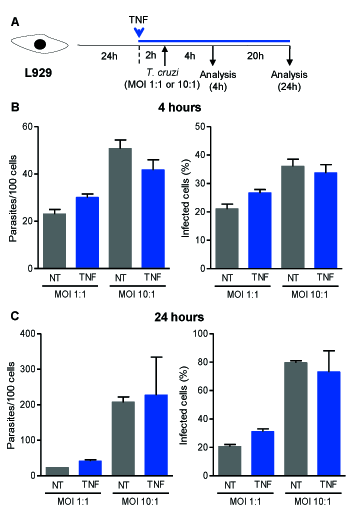

Supplement: Supplementary file 4 — Pre-treatment of fibroblast with rTNF does not affect infection by Trypanosoma cruzi. (a) Experimental scheme showing that L-929 fibroblasts were pre-treated with TNF (1 ng/mL) and subsequently infected with trypomastigote forms of the Colombian T. cruzi strain. (b-c) The percentage of infected cells and the number of parasites per cells were analyzed at 4 h (b) and 24 h (c) of infection, at a MOI of 1:1 or 10:1. Data are presented as mean ± SEM of duplicates. (TIFF 1430 kb) [file 12974_2017_952_MOESM4_ESM.tif]

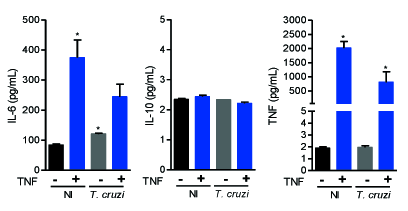

Supplement: Supplementary file 5 — Pre-exposing astrocyte cultures to rTNF enhances parasite load in Trypanosoma cruzi-infected astrocytes. C57BL/6 primary astrocyte cultures were seeded, exposed to rTNF and infected as described in Fig. 5a. At 24 and 48 h of infection, the frequencies of infected astrocytes were stratified by classes according to the number of amastigote-like forms harbored in the cytoplasm. Data are presented as means ± SEM of triplicates. *, p < 0.05, **, p < 0.01 untreated (NT) vs. rTNF-treated astrocytes. (TIFF 1045 kb) [file 12974_2017_952_MOESM5_ESM.tif]

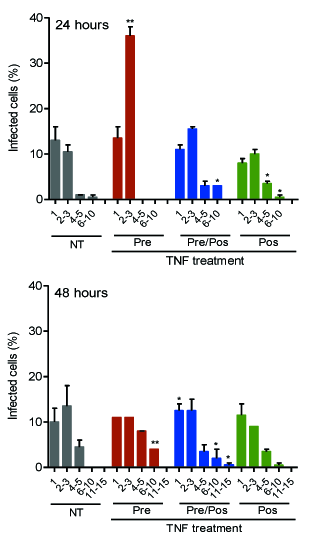

Supplement: Supplementary file 6 — rTNF amplifies the pro-inflammatory profile of human astrocytes exposed to Trypanosoma cruzi infection. Human astrocytes were submitted to treatment with rTNF and left non-infected (NI) or infected by T. cruzi (MOI 1:1). At 4 h of infection, supernatants were collected and submitted to detection of cytokines by CBA. Data are presented as means ± SEM of triplicates. *, p < 0.05, untreated (NT) vs. rTNF-treated astrocytes. (TIFF 1375 kb) [file 12974_2017_952_MOESM6_ESM.tif]

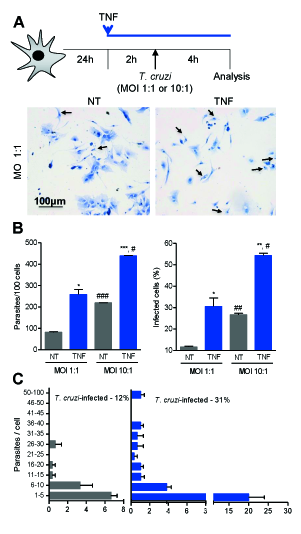

Supplement: Supplementary file 7 — rTNF effects on Trypanosoma cruzi/astrocyte interaction are conserved in primary astrocyte cultures of C3H/He mice. (a) Monotypic primary astrocyte cell cultures of C3H/He mice were seeded, left untreated or treated with rTNF and infected with trypomastigote forms of the Colombian T. cruzi strain at a MOI of 1:1 or 10:1. (b) After 4 h of interaction, the frequencies of infected cells and the number in untreated (NT), rTNF-treated astrocytes are shown. (c) Data show the frequencies of infected astrocytes stratified by classes according to the number of amastigote-like forms harbored in the cytoplasm. Data are presented as means ± SEM of triplicates. *, p < 0.05, ***, p < 0.001 untreated (NT) vs. rTNF-treated astrocytes. #, p < 0.05, ##, p < 0.01, ###, p < 0.001 MOI 1:1 vs. MOI 10:1. (TIFF 1337 kb) [file 12974_2017_952_MOESM7_ESM.tif]
